# Assessment of Gait Parameters Using Wearable Sensors and Their Association With Muscle Mass, Strength, and Physical Performance in Korean Older Adults: Cross-Sectional Study

**DOI:** 10.2196/63928

**Published:** 2025-04-10

**Authors:** Jinyoung Shin, Hyuk Jung Kweon, Jaekyung Choi

**Affiliations:** 1 Department of Family Medicine, Konkuk University Medical Center, Konkuk University School of Medicine, Seoul, Republic of Korea; 2 Department of Family Medicine, Konkuk University Medical Center, Chungju Hospital, Konkuk University School of Medicine, 268 Chungwon-daero, Chungju, 27376, Republic of Korea, 82 43 840 8200

**Keywords:** gait analysis, sarcopenia, wearable electronic devices, muscle mass, physical performance, older adults, geriatric, cross-sectional study, outpatient clinic, Korea, mHealth, mobile health

## Abstract

**Background:**

Gait speed indicates the onset or decline of physical performance in sarcopenia. However, real-time measurements of other gait parameters, such as step length, stride length, step width, and support time, are limited. The advent of wearable technology has facilitated the measurement of these parameters, necessitating further investigation into their potential applications.

**Objective:**

This study aimed to investigate the relationship between gait parameters measured using wearable sensors and muscle mass, strength, and physical performance in community-dwelling older adults.

**Methods:**

In a cross-sectional study of 91 participants aged ≥65 years, gait parameters, such as step count, step length, cadence, single and double support times, vertical oscillation, and instantaneous vertical loading rate (IVLR), measured using a wireless earbud device, were analyzed on the basis of the appendicular skeletal muscle mass index (SMI), calf circumference, handgrip strength, 5-time chair stand test, short physical performance battery (SPPB), and the SARC-F (strength, assistance with walking, rise from a chair, climb stairs and fall frequency) questionnaire. This study was conducted from July 10 to November 1, 2023, at an outpatient clinic of a university hospital in Seoul, Korea. Multiple regression analysis was performed to investigate independent associations after adjusting for age, sex, BMI, and comorbidities.

**Results:**

Among 91 participants (45 men and 46 women; mean age 74.1 years for men and 73.6 years for women), gait speed and vertical oscillation showed negative associations with their performance in the 5-time chair stand test (*P*<.001) and SARC-F and positive associations with their performance in the SPPB (*P*<.001). Vertical oscillations were also associated with grip strength (*P*=.003). Single and double support times were associated with performance in the 5-time chair stand test and SPPB (*P*<.001). In addition, double support time was associated with SARC-F scores (*P*<.001). Gait speed, support time, vertical oscillation, and IVLR showed independent associations with performance in the 5-time chair stand test and SPPB (*P*<.001), both related to muscle strength or physical performance. Gait speed, double support time, and vertical oscillation all had significant associations with SARC-F scores.

**Conclusions:**

This study demonstrated a significant association between gait monitoring using wearable sensors and quantitative assessments of muscle strength and physical performance in older people. Furthermore, this study substantiated the extensive applicability of diverse gait parameters in predicting sarcopenia.

## Introduction

Sarcopenia is an age-related condition characterized by the progressive loss of skeletal muscle mass and strength, as well as physical performance [1], which increases the risk of disability, falls, cognitive decline, loss of independence, and mortality among community-dwelling older adults [2-5]. The International Clinical Practice Guidelines for Sarcopenia recommend annual clinical assessments or more frequent evaluations after major health events, such as hospitalization, to detect early signs of frailty [6,7]. According to the 2019 Asian Working Group for Sarcopenia (AWGS) consensus update, the diagnosis of sarcopenia should be based on changes in muscle strength, physical performance, and muscle mass, with case findings leading to a comprehensive assessment and diagnosis in primary care or community preventive settings [8-10].

Gait monitoring, particularly gait speed, has been identified as a valid indicator of sarcopenia and is associated with health outcomes such as disability and mortality in older adults [11,12]. Although various gait parameters such as step length, stride length and width, and single support time during walking can be detected, their use in research has been limited owing to challenges in real-time measurement and result standardization [13]. Wearable sensors have made it easier to conduct real-time evaluations. A meta-analysis of 82 studies addressing over 100 gait parameters outcomes discovered high validity and reliability, particularly for spatiotemporal parameters such as step time and stride time [14].

Because smartphone apps enable the measurement of spatiotemporal outcomes while older adults walk, the clinical utility of real-time monitoring of changes in mobility among older adults using wearable sensors has been highlighted [15]. Daily walking count guidance using wearable sensors improves physical activity and reduces sarcopenia in older adults in Taiwan [16]. A study examining men older than 50 years in Korea using a wearable smart belt for 4 weeks discovered significant correlations between gait speed and sarcopenia, as defined by grip strength and dual-energy x-ray absorptiometry [17]. Several studies measured gait parameters using watches or footwear [18]. However, few studies have investigated the association between various gait parameters other than gait speed or step length and sarcopenia diagnosis based on muscle strength, muscle mass, and physical performance using an earbud device [19,20]. This study aimed to investigate the relationship between various gait parameters measured using wearable sensors (particularly earbud-type sensors) and sarcopenia, based on muscle mass, strength, and physical performance, among community-dwelling older adults.

## Methods

### Study Design and Participants

This is a cross-sectional study. The primary objective of this study was to investigate the association between gait parameters and various sarcopenia measurements such as muscle mass, strength, and physical performance. The secondary objective was to determine if newly usable gait parameters, which can be easily measured using wearable sensors, were significantly associated with sarcopenia. In the initial study, the target sample size for both men and women was 45 individuals in an initial study [21]. This analysis was performed as a secondary study, using data from a comparison study of a portable device for muscle mass and bioelectrical impedance analysis (BIA). At an older adult community center, we recruited 91 participants aged 65 years or older who could perform daily activities independently. We displayed a recruitment poster at the community center, inviting older adults interested in the study to apply to participate. This study was performed at the outpatient clinic of a university hospital in Seoul, Korea, between July 10 and November 1, 2023. The exclusion criterion was ongoing treatment for uncontrolled conditions such as malignancy, acute stroke, or dementia.

### Muscle Mass, Strength, and Physical Performance Measurements

Muscle mass was determined using the appendicular skeletal muscle mass index (SMI) and calf circumference. The SMI is calculated by dividing the total lean mass of both arms and legs by the square of the height (kg/m^2^) as measured using BIA (InBody 770; Cerritos). Calf circumference was measured at the thickest point on both sides, with moderate to high sensitivity and specificity for detecting low skeletal muscle mass [10,22]. Muscle strength was determined by the grip strength measured 3 times at 30-second intervals for each hand in a standing position, alternating between the dominant and nondominant sides, using a Smedley handheld dynamometer [23]. The 5-time chair stand test, which assesses both physical performance and muscle strength, requires participants to stand and sit 5 times as quickly as possible from a straight-backed chair with no arm support [24]. A short physical performance battery (SPPB) was used to assess balance, gait, and lower limb strength. This included standing with feet side-by-side, semitandem, and tandem positions; walking 8 feet; and rising from a chair to return to a seated position 5 times [25]. The SARC-F (strength, assistance with walking, rise from a chair, climb stairs and fall frequency) questionnaire was used to assess sarcopenia risk based on its 5 components, with fall frequency ranging 0-10. A total score of ≥4 indicates sarcopenia. The Korean version of the SARC-F was previously validated [26,27]. According to the AWGS consensus report in 2014, we used the cutoff values for SMI (less than 7.0 kg/m^2^ for men and less than 5.7 kg/m^2^ for women using BIA) and calf circumference (<34 cm for men and <33 cm for women) [1]. Low muscle strength was defined as grip strength of <28 kg for men and <18 kg for women. The cutoff value for the 5-time chair stand test was 12 seconds or more, while that of the SPPB was ≤9 (range 0‐12) points [10].

### Gait Parameters

**Figure 1.**
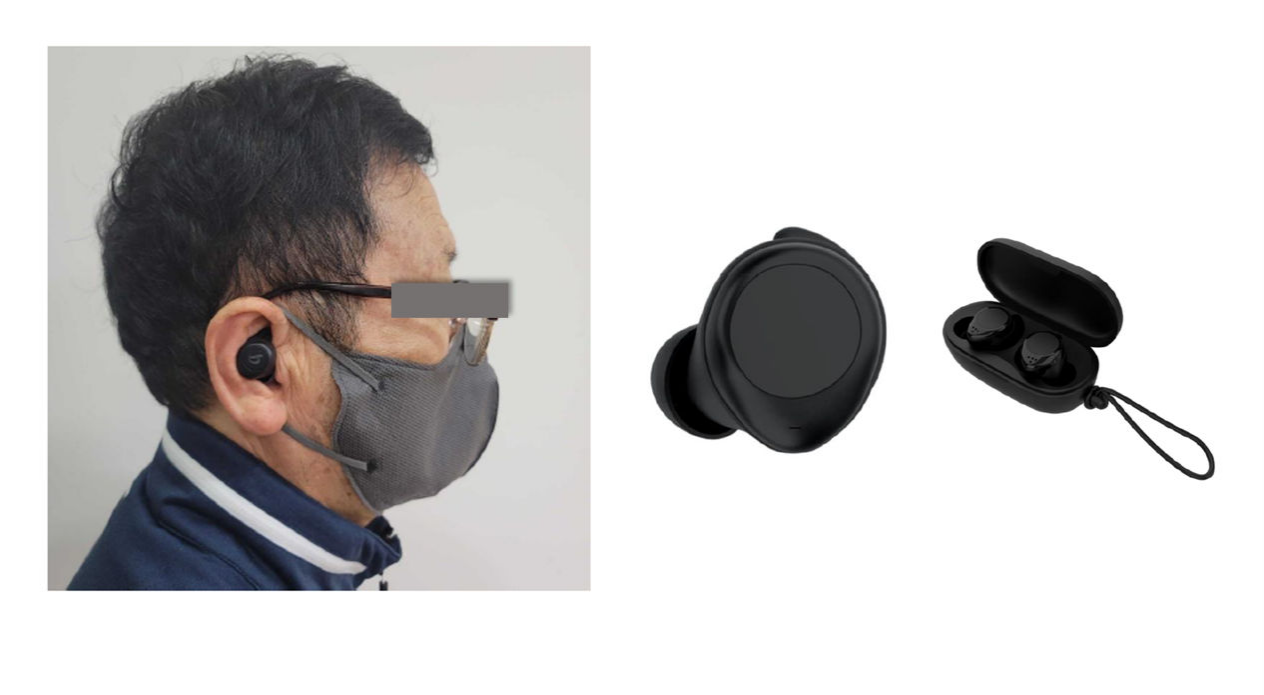
Wearable sensors for gait monitoring (5.7 g, 17.1 × 20.2 × 22 mm^3^).

Gait characteristics were measured using a wireless earbud (Beflex) device (Figure 1) equipped with a BiomechEngine chip for accurate accelerometer data analysis and gait parameter estimation. In young adults, the earbud device’s validity was confirmed by measuring walking and running parameters [28]. Participants were instructed to insert the device into both ears and walk for a minimum of 30 seconds to record various gait metrics such as step count, step length, cadence, gait speed, times of single and double support, vertical oscillation, and instantaneous vertical loading rate (IVLR). Step count is defined as the number of steps analyzed within a 2-second interval, where a step from the left foot to the right foot is considered a single cycle of 2 steps. Step length was calculated as the distance between the heel of the front foot and heel of the back foot during the walking cycle. Gait speed and cadence were automatically calculated. Single support time was defined as the average duration of one foot supporting the body while walking. Double support time was defined as the average duration of both feet’s support and vertical oscillation, which was defined as the average distance of the body’s center moved vertically during walking [28]. The IVLR measures the steepest slope of the vertical ground reaction force at the points of left- and right-foot contact. The algorithms used to estimate these gait parameters are not currently disclosed; however, they are commercially available. Gait parameters were measured a few minutes after walking.

### Ethical Considerations

The study was conducted according to the guidelines of the Declaration of Helsinki, and approved by the institutional review board of Konkuk University Medical Center (KUMC 2022-11-033). Informed consent was obtained from all subjects enrolled in the study. Written informed consent was obtained from the patients for publication of this paper. The authors attest that there was no use of generative artificial intelligence technology in the generation of text, figures, or other informational content in this manuscript.

### Statistical Analysis

Descriptive statistics were used to summarize participants’ characteristics based on sex. Independent samples 2-tailed *t* tests were used to compare continuous variables, whereas chi-square tests were used for categorical variables. Pearson correlation analysis was used to determine the relationship between gait parameters and sarcopenia. We performed a logarithmic transformation to measure gait, muscle, and physical performance to adjust for a skewed distribution. After adjusting for age, sex, BMI, and comorbidities, multiple regression analysis was used to investigate the independent associations between sarcopenia and specific gait parameters for the secondary end point. Participants reported their age, sex, and comorbidities, as well as any daily life difficulties caused by low vision or hearing impairment (“yes” or “no”). Height and weight were measured to calculate the BMI (kg/m^2^) and waist circumference at the narrowest point. Bonferroni correction for the evidential threshold was performed based on the number of exposures for the 6 sarcopenia indices; therefore, statistical significance was set at *P*<.05 divided by 6 (ie, a threshold of *P*<.0083 per exposure). All statistical analyses were conducted using IBM SPSS Statistics (version 27.0).

## Results

This study included 91 participants (45 men and 46 women). The participants’ characteristics are summarized in Table 1. The mean age of the study participants was 73.9 years, with more than half of them aged between 65 and 74 years. According to Asian criteria for overweight and obesity, the participants had a normal BMI and waist circumference [29].

While there were differences in grip strength, calf circumference, and SMI between men and women, there were no differences in their performance in the 5-time chair stand test, SPPB, or SARC-F (Table 2). The prevalence of sarcopenia varied according to the definition used for each measurement, ranging from 4.3% (SARC-F) to 82.6% (5-time chair stand test). The prevalence of sarcopenia according to grip strength was higher in women than in men. However, the prevalence of sarcopenia, defined by the SMI, was higher in men than in women. There were no in the other indicators between men and women.

Table 3 lists the association between various gait parameters and sarcopenia measurements. Gait speed, single and double support times, vertical oscillation, and IVLR demonstrated significant correlations with performance on the 5-time chair stand test and the SPPB. Additionally, gait speed, double support time, and vertical oscillation were associated with performance on the SARC-F. Vertical oscillation positively correlated with grip strength, and step count negatively correlated with performance on the 5-time chair stand test. However, after Bonferroni correction, step length and cadence did not show any significant correlations with sarcopenia. Calf circumference and SMI were not associated with gait parameters.

After adjusting for confounding factors, gait speed, double support time, and vertical oscillation were significantly correlated with the 5-time chair stand test, SPPB, and SARC-F scores (Table 4). Single support time and IVLR were significantly correlated with performance in the 5-time chair stand test and SPPB.

**Table 1.** Characteristics of participants.

		Total (n=91)	Men (n=45)	Women (n=46)
Age (years), mean (SD)		73.9 (5.5)	74.1 (5.0)	73.6 (6.1)
**Age groups (years), n (%)**				
	65‐74	51 (56.0)	24 (53.3)	27 (58.7)
	75‐84	38 (41.8)	21 (46.7)	17 (37.0)
	≥85	2 (2.2)	0 (0)	2 (4.3)
BMI (kg/m^2^), mean (SD)		24.4 (2.8)	24.6 (2.5)	24.2 (3.1)
Waist circumference (cm), mean (SD)		80.0 (8.9)	83.0 (7.4)	77.0 (9.3)
Number of comorbidities, mean (SD)		1.46 (1.07)	1.58 (0.97)	1.35 (1.16)
**Comorbidities, n (%)**				
	None	17 (18.7)	5 (11.1)	12 (26.1)
	Cardiovascular disease	69 (75.8)	40 (88.9)	29 (63.0)
	Musculoskeletal disease	14 (15.4)	2 (4.4)	12 (26.1)

**Table 2.** Diagnostic measurements of sarcopenia in study participants.

	Measurements			Sarcopenia		
	Men (n=45), mean (SD)	Women (n=46), mean (SD)	*P* value	Men (n=45), n (%)	Women (n=46), n (%)	*P* value
5-time chair stand test^a^	15.7 (5.5)	15.7 (4.6)	.96	33 (73.3)	38 (82.6)	.29
SPPB^b^	9.0 (1.5)	8.9 (1.5)	.73	29 (64.4)	29 (63.0)	.89
SARC-F^c^	0.66 (1.51)	0.87 (1.34)	.50	3 (6.7)	2 (4.3)	.63
Grip strength^d^	28.8 (5.6)	18.1 (4.8)	<.001	13 (28.9)	23 (50.0)	<.001
Calf circumference^e^	34.2 (2.8)	32.1 (2.5)	<.001	20 (44.4)	25 (54.3)	.35
Skeletal muscle index^f^	7.7 (0.71)	6.2 (0.6)	<.001	7 (15.6)	5 (10.9)	.02

a Cutoff was ≥12 seconds.

b SPPB: short physical performance battery; cutoff was ≤9 points.

c SARC-F: strength, assistance with walking, rise from a chair, climb stairs and fall frequency; cutoff was ≥4 points.

d Cutoff was <28 kg for men and <18 kg for women.

e Cutoff was <34 cm for men and <33 cm for women.

f Cutoff was <7.0 kg/m2 for men and <5.7 kg/m2 for women.

**Table 3.** Associations between gait parameters and sarcopenia measurements (n=91). All variables were subjected to correlation analysis after log transformation and Bonferroni correction was applied (*P*<.05/6 [.0083]).

		5-time chair stand test	SPPB^a^	SARC-F^b^	Grip strength	Calf circumference	SMI^c^
**Step count**							
	Coefficient	−0.322	0.163	−0.137	−0.071	−0.198	−0.204
	*P* value	.002	.12	.20	.50	.06	.053
**Step length**							
	Coefficient	−0.221	0.237	−0.209	−0.162	−0.176	−0.162
	*P* value	.04	.02	.047	.13	.10	.13
**Cadence**							
	Coefficient	−0.018	−0.036	−0.191	0.057	−0.041	0.040
	*P* value	.86	.75	.07	.59	.70	.71
**Gait speed**							
	Coefficient	−0.437	0.381	−0.281	−0.176	−0.196	−0.230
	*P* value	<.001	<.001	.007	.10	.06	.03
**Single support time**							
	Coefficient	0.489	−0.350	0.176	0.167	0.158	0.255
	*P* value	<.001	.001	.10	.11	.14	.02
**Double support time**							
	Coefficient	0.604	−0.541	0.371	−0.007	0.083	0.151
	*P* value	<.001	<.001	<.001	.95	.43	.15
**Vertical oscillation**							
	Coefficient	−0.387	0.518	−0.467	0.306	0.107	0.198
	*P* value	<.001	<.001	<.001	.003	.31	.06
**IVLR^d^**							
	Coefficient	−0.530	0.449	−0.270	−0.032	−0.128	−0.177
	*P* value	<.001	<.001	.01	.76	.23	.09

a SPPB: short physical performance battery.

b SARC-F: strength, assistance with walking, rise from a chair, climb stairs and fall frequency.

c SMI: appendicular skeletal muscle mass index.

d IVLR: instantaneous vertical loading rate.

**Table 4.** Multiple regression analysis for sarcopenia and gait indexes in older adults, having adjusted for age, sex, waist circumference, BMI, and comorbidities. Bonferroni correction was applied (*P*<.05/6 [.0083]).

	5-time chair stand test		SPPB^a^		SARC-F^b^	
	Coefficient	*P* value	Coefficient	*P* value	Coefficient	*P* value
Gait speed	−0.393	<.001	0.331	.001	−0.328	.003
Single support time	0.545	<.001	−0.384	.001	0.222	.055
Double support time	0.558	<.001	−0.452	<.001	0.389	<.001
Vertical oscillation	−0.306	.008	0.399	<.001	−0.528	<.001
IVLR^c^	−0.496	<.001	0.392	<.001	−0.267	.02

a SPPB: short physical performance battery.

b SARC-F: strength, assistance with walking, rise from a chair, climb stairs and fall frequency.

c IVLR: Instantaneous vertical loading rate.

## Discussion

### Principal Findings

Gait measurements using wearable sensors show a strong correlation with physical performance and muscle strength. This study confirmed that the gait speed, double support time, and vertical oscillation measured using wearable sensors were reliable walking indicators of sarcopenia in community-dwelling older adults. Furthermore, using real-time gait monitoring measurements, we found that single support time and IVLR were good indicators of sarcopenia.

Age-related alterations in gait, such as shorter steps, increased double support time, lower cadence, or a wider step width, indicate a decline in gait speed [30]. These changes are attributed to a combination of sensorimotor factors, including motor unit reduction, impaired muscular activation, fiber-type substitution, decreased cutaneous sensation, and slower nerve and reaction speeds [12]. Furthermore, functional brain impairment, manifested as reduced brain volume, may be associated with deterioration in gait performance [31]. Accordingly, among the diagnostic indicators of sarcopenia, gait parameters may show a high correlation with the physical performance of participants.

However, these gait parameters were not associated with calf circumference or SMI, which are indicators of muscle mass. BIA, combined with the measurement of anthropometric metrics such as calf circumference, provides a practical approach for estimating muscle mass in nonclinical settings [11]. Individual factors, such as edema or diuretics may influence BIA [32], and muscle mass changes in older adults are not uniform [22], necessitating caution when interpretating their correlation with gait parameters. However, it is important to note that muscle mass is not a good predictor of adverse health–related outcomes such as mobility limitation, falls, and mortality in community-dwelling older adults [9]. Therefore, sarcopenia in older adults should be diagnosed holistically with muscle strength and physical performance taking precedence over muscle mass. Although this study did not identify a universal correlation between handgrip strength and gait parameters, quadriceps strength has been shown to influence gait speed, step length, and cadence [30]. This emphasizes the limitations of handgrip strength as an indicator of overall muscle strength in healthy older adults [33,34]. Further studies are required to determine the association between upper and lower limb muscle strength and gait parameters.

Among the gait parameters, gait speed, either alone or as part of a combined tool, such as the SPPB, has been confirmed to be associated with sarcopenia, which is the most comprehensive indicator of brain function and structural abnormalities [35]. Double support time correlates with balance in the cortico-subcortical white matter sensory and motor tracts [35]. Gait speed and double support time are direct indicators obtained while walking, whereas vertical oscillation and IVLR are known to be sensitive in reflecting changes in brain function as indirect data derived from direct indicators [36]. We confirmed the independent association between vertical oscillation and sarcopenia. Therefore, analyzing various gait parameters could improve the accuracy of sarcopenia diagnosis in older adults. Gait measurement using wearable sensors, which are easy to use and allow continuous and periodic monitoring of daily activities, offers significant promise for the early detection of sarcopenia in both the home and caregiving facilities. In general, older adults have difficulty with consistent use on consecutive days. Therefore, compliance with wearable sensors must be considered when evaluating the effectiveness of wearables in monitoring physical activity in older populations [37].

### Limitations

However, several limitations of this study should be considered when applying gait measurements to older adults. First, the majority of participants were 65-74 years old, relatively healthy, and capable of independent daily living; hence, our results may not be applicable to all older populations. Additional research is required to apply this device to older adults living in nursing homes or other specialized care facilities. Second, we were unable to compare differences in wearable sensor techniques because our study used earbud-type devices rather than those more commonly placed on the foot or shank or used an insole pressure-sensing method [38]. We did not conduct repeated tests to assess test-retest reliability because it was difficult to revisit older participants. Third, because this study was conducted using a cross-sectional design, we could not establish a temporal relationship between the deterioration of gait parameters and sarcopenia. It is important to note that differences in the prevalence of sarcopenia based on the definition of muscle mass, strength, and physical performance may influence the correlation with gait parameters. Specifically, because our study did not compare gait parameters based on different rates of sarcopenia diagnosis, clinical interpretation should be cautious when predicting sarcopenia directly. Because race or lifestyle habits may have affected our results, it is important to consider the characteristics of the participants when applying the findings of this study. Finally, single and double support times, vertical oscillation, and IVLR still have limitations in practical applications, because diagnostic criteria have yet to be established. The algorithms for estimating these gait parameters are not currently disclosed; therefore, further studies are required to identify the clinical cutoff points to predict sarcopenia.

### Conclusions

Using earbud-type wearable sensors, this study confirmed that gait parameters, particularly gait speed, double support time, and vertical oscillation, are correlated to muscle strength and physical performance in community-dwelling older adults. Given the limitations associated with the current diagnostic criteria and the potential unreliability of single measurements, further research is imperative to establish efficacy measures and broaden the applicability of diverse gait parameters in sarcopenia diagnosis.

## Abbreviations

AWGS: Asian Working Group for Sarcopenia
BIA: bioelectrical impedance analysis
IVLR: instantaneous vertical loading rate
SARC-F: strength, assistance with walking, rise from a chair, climb stairs and fall frequency
SMI: appendicular skeletal muscle mass index
SPPB: short physical performance battery
